# Korean Cattle 3D Reconstruction from Multi-View 3D-Camera System in Real Environment

**DOI:** 10.3390/s24020427

**Published:** 2024-01-10

**Authors:** Chang Gwon Dang, Seung Soo Lee, Mahboob Alam, Sang Min Lee, Mi Na Park, Ha-Seung Seong, Seungkyu Han, Hoang-Phong Nguyen, Min Ki Baek, Jae Gu Lee, Van Thuan Pham

**Affiliations:** 1 National Institute of Animal Science, Rural Development Admission, Cheonan 31000, Republic of Korea; gkgkgki@korea.kr (C.G.D.); genemap@korea.kr (S.S.L.); mahboob@korea.kr (M.A.); iq6000@korea.kr (S.M.L.); mina0412@korea.kr (M.N.P.); seonghs07@korea.kr (H.-S.S.); 2 ZOOTOS Co., Ltd., R&D Center, Anyang 14118, Gyeonggi-do, Republic of Korea; lion@zootos.com (S.H.); hippo@zootos.com (H.-P.N.); bear@zootos.com (M.K.B.)

**Keywords:** Korean cattle, point cloud registration, 3D segmentation, AI

## Abstract

The rapid evolution of 3D technology in recent years has brought about significant change in the field of agriculture, including precision livestock management. From 3D geometry information, the weight and characteristics of body parts of Korean cattle can be analyzed to improve cow growth. In this paper, a system of cameras is built to synchronously capture 3D data and then reconstruct a 3D mesh representation. In general, to reconstruct non-rigid objects, a system of cameras is synchronized and calibrated, and then the data of each camera are transformed to global coordinates. However, when reconstructing cattle in a real environment, difficulties including fences and the vibration of cameras can lead to the failure of the process of reconstruction. A new scheme is proposed that automatically removes environmental fences and noise. An optimization method is proposed that interweaves camera pose updates, and the distances between the camera pose and the initial camera position are added as part of the objective function. The difference between the camera’s point clouds to the mesh output is reduced from 7.5 mm to 5.5 mm. The experimental results showed that our scheme can automatically generate a high-quality mesh in a real environment. This scheme provides data that can be used for other research on Korean cattle.

## 1. Introduction

In general, to reconstruct 3D objects, people can use 2D images or 3D cameras. Using 2D images as input, we can reconstruct objects like buildings by the Structure From Motion algorithm [1], whereas 3D cameras have an advantage in reconstructing objects in a shorter range due to the high-quality data captured like the Kinect Fusion program [2]. In the object reconstruction field by 3D cameras, objects are simply separated into two types: rigid objects and non-rigid objects.

For rigid objects, a sequence of frames is captured by one camera, then global registration is implemented [2,3]. When two frames are closed and have large overlaps, the ICP algorithm [4,5,6,7,8] can be used for registering between frames. In the case of initial poses between two cameras that are unknown, Rusu in 2009, proposed a method of global alignment by global feature FPFH [9]. After locally aligning pairs of frames, the positions of all frames are aligned locally by the pose graph algorithm [10] or loop closure [2].

In the case of reconstruction of non-rigid objects, using one camera to reconstruct [11,12] a moving non-rigid object has many limits due to the fast change of non-rigid objects. In general, to reconstruct non-rigid objects, a system of cameras needs to be synchronized and calibrated and then data from each camera are transformed to global coordinates.

However, when reconstructing cattle in a real environment, difficulties, including occlusion by surrounding fences and the vibration of cameras, can lead to the failure of the process of reconstruction. The camera position can be vibrated by wind or ground vibration. Thus, although the camera system is calibrated beforehand, the camera’s point cloud cannot be directly mapped to global coordinates to form a well-defined 3D shape. Environmental fences, which partially prevent data capture, create large empty areas on the cow’s surface and generate noisy data in areas near fences. The camera vibration and fences make the reconstruction a challenging task.

**Figure 1:**
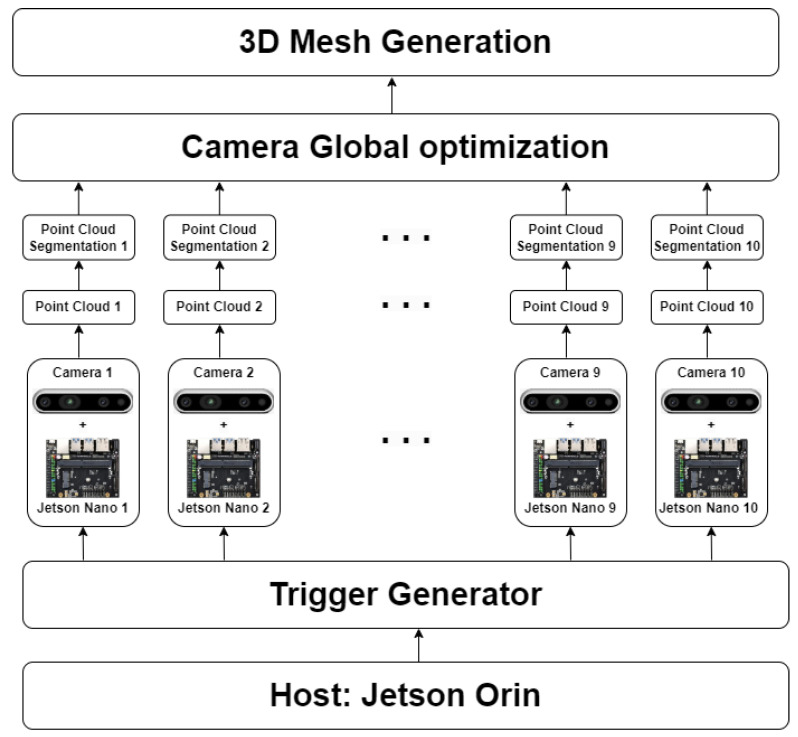
The Korean cattle 3D reconstruction framework.

Therefore, we proposed an integrated system to overcome the above disadvantages. The system is divided into two main parts. The hardware part: A system of ten cameras (Figure 1) are calibrated and synchronously capture stereo images. The application part: cow surface segmentation and camera pose optimization are implemented. The camera’s initial pose is established based on the calibration result. Each camera is optimized to well overlap with other cameras but needs to not be far from the original pose. The optimization result is compared to the global optimization method, BCMO [13]. The contributions of this paper can be summarized as follows:

1. A method of two-step optimization is proposed. In step 1, a global mesh is generated from all cameras, and in step 2, each camera is aligned to the global mesh. This optimization can run well with cameras that have small overlapping and considerable noise.
2. Automatic 3D segmenting of cattle parts from 3D data of each camera.

The structure of the rest of this paper is organized as follows: In Section 2, we present papers related to global registration; in Section 3, are the materials and methods that provide details of the scanning system and the data acquisition process. Section 4 focuses on the segmentation of data for each camera, and the optimization of the global camera pose is mentioned. Section 5 presents the experimental results that include the evaluation of time synchronization evaluation of the optimization process. The comparison of global optimization between our result and the result of the BCMO algorithm is present. Finally, Section 6 is the conclusions and future work.

## 2. Related Work

Recently, reconstructing animals, such as cattle, has emerged as an appealing research topic. One can use a single 3D scan camera to capture a sequence of frames and then apply global alignment [2,14,15]. However, a drawback of this method is that it requires the cattle to be still. Reconstruction of non-rigid objects is a developing topic, and this method can reconstruct a small movement of non-rigid objects. Applying this reconstruction method to reconstruct cattle is not practical because of the fast movement of animals.

Another reconstruction method is using multi-view cameras. A system of cameras is built to synchronously scan data and then align all frames to create the point cloud [16,17,18,19]. However, in paper [16,17], the fence size must be small, or the registration algorithm used (Super4PCS) can not work with small overlapping point clouds. In paper [20], they used laser scanners to generate high-quality point clouds but only reconstructed non-moving cows. Camera synchronization is essential in this method. The iterative closest point [21] is known as a widely used algorithm to determine alignment between two roughly aligned 3D point clouds. This algorithm searches for correspondence points between two given point cloud sets and then optimizes object functions with the aim of minimizing the distance between corresponding points. However, if the overlap between a pair of cameras is small or there is no data because of occlusion from fences leading to alignment by ICP, the algorithm can fail. The global alignment method for the cloud from all cameras in paper [18,19] used information on building a set of co-planar points to register.

A pre-trained model, CreStereo [22], is applied to enhance the depth quality [23], and our approach similarly leverages this model to improve the reconstruction result. The camera’s position is calibrated by spheres [24], and its calibration information is used for the reconstruction process. All of our cameras are calibrated before reconstruction so we can obtain an initial position of all cameras. Different from the global optimization method by the 4PCS or ICP algorithm, a global mesh is created from all input cameras, and then a process of two steps is repeated. Step one is aligning all cameras to the global mesh and step 2 is to compute the global mesh from all cameras. In step 1, the objective function is modified from the ICP algorithm that guarantees the camera position is not far from the initial position. This method prevents cameras from sliding away from the initial position.

## 3. Material and Data Acquisition

### 3.1. The 3D Reconstruction Framework

In this section, we proposed a 3D reconstruction scheme that generates a cattle 3D point cloud and registration issues. As indicated in Figure 1, the 3D reconstruction framework contains three main parts:

- Camera synchronization and data acquisition.
- Segmenting 3D data.
- Global 3D camera pose optimization and generating mesh.

In this system, we can generate a full cow mesh. The Host, Jetson Orin, is the center of the system; it connects to a trigger generator to synchronously capture data from ten cameras. Data from ten cameras are transferred to Orin Host through a Wi-Fi connection. The processes of point cloud generation, segmentation, camera global optimization, and mesh generation run on Host Orin.

### 3.2. Cameras System

Our camera system includes:

- Trigger Generator: This device generates a synchronous signal to ten camera devices.
- Camera devices—Intel Realsense D435i: This device connects and sends stereo images to Jetson Nano.
- Single Board Computer—Jetson Nano: This device receives stereo images from cameras and then transfers them to Jetson Orin.
- Host Computer—Jetson Orin: Center device of capturing system. It generates a signal to the active Trigger Generator. It retrieves stereo images from Jetson Nano devices and reconstructs the final 3D mesh.

**Figure 2:**
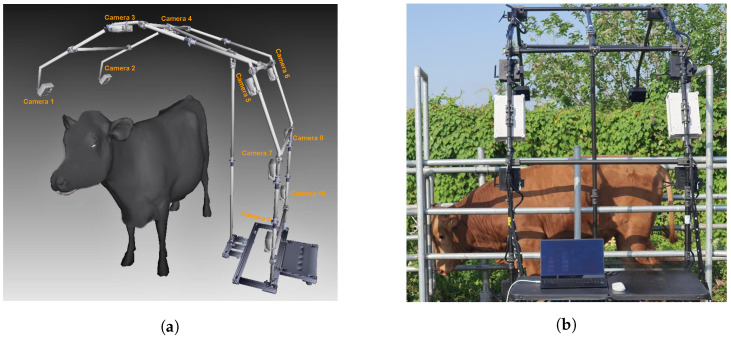
(**a**) Design of multi-view camera system. (**b**) Real multi-view camera system.

Figure 2a is a rendering of the camera system model and Figure 2b is an image of the camera system at the farm. The camera system can be easily installed, dismantled, and moved, but it is susceptible to being shaken by the environment or the wind.

The device specifications are detailed in Table 1.

### 3.3. Camera Synchronization System

A multi-camera synchronization system has been developed to register frames acquired from ten cameras. We use Trigger TG-16 to control all cameras. The trigger device synchronously sends a capturing signal to 10 camera devices to capture cattle data. Additionally, synchronization global timestamps among host Orin and other Jetson Nano devices are set up based on Network Time Protocol. Consequently, we can retrieve simultaneously captured frames by gathering each frame from all cameras that share the same global timestamps.

### 3.4. Camera Calibration System

Calibration consisted of finding the camera’s intrinsic parameters and the global position of each camera. The cameras were calibrated in two ways:

- Calibration of each camera to determine its intrinsic parameters.
- Calibration global extrinsic matrix of all ten cameras.

A system of spheres is designed for calibration. After capturing data, the center of all spheres on each camera can be detected. The calibration system is designed with the distance between spheres being different so we can find correspondence of a sphere’s center points on the point cloud of all cameras. We calibrated all pairs of cameras to find calibration information on all cameras. Calibration was performed twice, first in the laboratory and then after installation on the farm. The camera matrix from the calibration process can be used as an initial camera’s position in the camera pose optimization process.

## 4. Korean Cattle 3D Reconstruction

### 4.1. Point Cloud Generation and Removing Fence for a Single Camera

#### Point Cloud Generation and Segmentation

**Figure 3:**
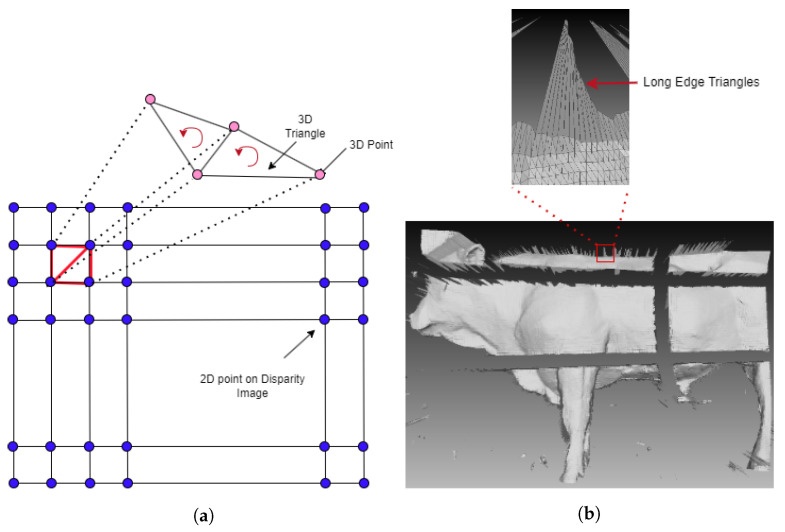
(**a**) The 3D mesh triangulation. (**b**) Mesh with long edges triangle.

In this work, the pre-trained model was based on the approach [22] for creating a disparity image from stereo images. In our system, we used images at a resolution of 1280 pixels by 720 pixels. A point cloud was generated by disparity image and camera intrinsic parameters as in Figure 3a. We considered a disparity image as a grid, and from 3 pixels side-by-side and not co-liner we back-projected to generate 3D points and then created a counter-clock-wise triangle. For each point, its normal is equal to the average normal of all triangles which contain that point. Points and normals were used for generating the 3D mesh.

**Figure 4:**
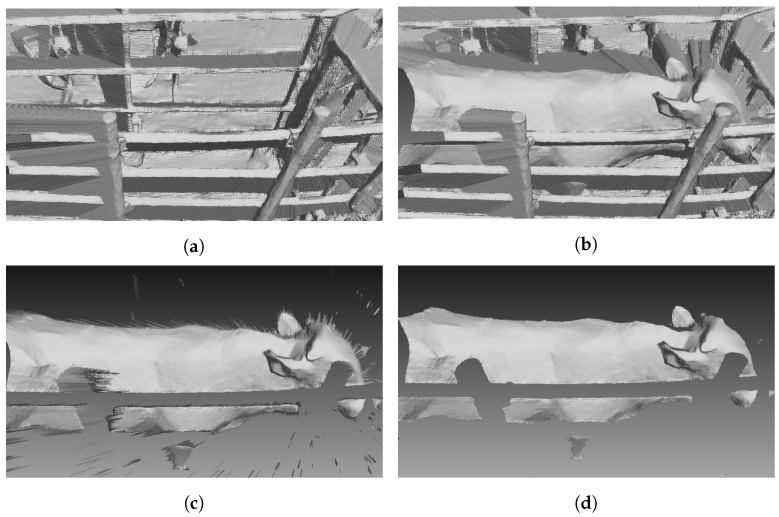
Point cloud generation and segmentation. (**a**) Environment mesh. (**b**) Cattle in environment mesh. (**c**) Environment removed mesh. (**d**) Cattle segmentation mesh.

Figure 4 shows a mesh segmentation process. Firstly, for all cameras, we created a 3D mesh of the environment without cattle, Figure 4a. A mesh of the environment with cattle is in Figure 4b. The mesh was generated by 3D mesh triangulation as in Figure 3a. All points of the environment in Figure 4b were removed to roughly obtain a mesh of cattle, like in Figure 4c. Points near areas in which depth has significantly changed can create noise triangles with long edges like in Figure 3b. A triangle that has a normal vector parallel to the camera direction has small edges. If a triangle’s normal vector and camera direction form a large angle, then that triangle has long edges. The average edge of a mesh is computed from all edges of the mesh. An edge is considered a long edge if it is two times longer than the average of the mesh. Next is the detection of the loop boundary of all mesh segmentations. The edge is a half-edge if it is contained in only one triangle. Connecting all half-edges to create a loop, a boundary of segmentation is created. The library, The Visualization Toolkit (VTK), is used to detect half-edges. To remove the remaining noise, mesh boundaries are detected and then deleted. In practice, the boundary is detected and deleted several times to remove all noise. This process allows us to obtain a segmentation mesh as shown in Figure 4d. To improve the computation time, a Kd-Tree of a point cloud of the environment is created one time and is used as a reference to remove 3D environment points of all cattle 3D frames.

### 4.2. Camera Pose Optimization

In this section, we present the approach in which camera poses are optimized.

#### 4.2.1. Objective

Here, input is the set of 10 segmented meshes which contain points and normals with initial camera pose, Ci. From the points and normals of 10 cameras that were generated by the triangulation method, we combined it into global points and normals, then set it as an input to generate a 3D model M by Poisson surface reconstruction (PSR) [25,26]. For each camera points cloud, we find an overlapping area of $P_{i}$ with other C subset $P_{i}$ based on the threshold distance value.

For a transform with small rotation angle, we can parameterize *T* by a 6-vector $\xi =(\alpha_{i},\beta_{i},\gamma_{i},t_{0i},t_{1i},t_{2i})$ Equation (1) that represents an incremental transformation relative to $T_{i}^{k}$ . Here, $\left(t_{0i},t_{1i},t_{2i}\right)$ is translation, and ${\left(\alpha_{i},\beta_{i},\gamma_{i}\right)}^{T}$ can be interpreted as the rotation part. $T_{i}^{k+1}$ is thus approximated by a linear function of $\xi_{i}$ :

(1) $$T_{i}^{k+1}\approx \left(\begin{array}{cccc} 1 & -\gamma_{i} & \beta_{i} & t_{0i}\\ \gamma_{i} & 1 & -\alpha_{i} & t_{1i}\\ -\beta_{i} & \alpha_{i} & 1 & t_{2i}\\ 0 & 0 & 0 & 1 \end{array}\right)T_{i}^{k}$$

Our objective is to optimize the set of cameras transform *T*, with mesh *M* serving as auxiliary variables. We minimize the objective function following:

(2) $$E\left(T\right)=\sum_{i}\sum_{p\in P_{i}}\left(Dist\left(M,p\right)\right)+\sum_{i}\left(\Gamma \left(T_{i},T_{i0}\right)\right)$$

whereas

(3) $$Dist\left(M,p\right)=min_{Tri_{k}\in M}\left(Dist\left(p,Tri_{k}\right)\right))$$

and,

(4) $$\Gamma \left(T_{i},T_{i0}\right)=\Gamma \left(T_{i0}^{-1}*T_{i}\right)=\Gamma \left(\left[R|t\right]\right)$$

(5) $${\left[R|t\right]}_{i}\approx \left(\begin{array}{cccc} 1 & -r_{i0} & r_{i1} & t_{i0}\\ r_{i0} & 1 & -r_{i2} & t_{i1}\\ -r_{i1} & r_{i2} & 1 & t_{i2}\\ 0 & 0 & 0 & 1 \end{array}\right)$$

(6) $$\Gamma \left(T_{i},T_{i0}\right)=k_{r}*\left(r_{i0}^{2}+r_{i1}^{2}+r_{i2}^{2}\right)+k_{t}*\left(t_{i0}^{2}+t_{i1}^{2}+t_{i2}^{2}\right)$$

In the objective function, Equation (2), the first part $\sum_{i}\sum_{p\in P_{i}}\left(Dist\left(M,p\right)\right)$ is the error difference between the point cloud of one camera to the other camera’s point cloud. The second part $\sum_{i}(\Gamma \left(T_{i},T_{i0}\right)$ shows the distance of each camera position to the initial camera position of that camera. In the process of data acquisition, the camera position can be rotated and translated. The camera translation can be large but the angle of rotation is generally small so we can approximate the second part of the Equation (5). Thus, the optimization process minimizes the error among the point cloud of all cameras but still keeps the camera position not moving far from the initial position. $k_{r}$ and $k_{t}$ are constant factors chosen at the beginning of the optimization process.

#### 4.2.2. Optimization

Here, we present an optimization scheme for minimizing the objective Algorithm 1. The optimization process is repeated in some iterations. In each iteration, the optimization is divided into 2 steps. In step 1, the global mesh is fixed, and then all 10 camera poses are optimized to the global mesh. In step 2, all camera poses are fixed and the global mesh is computed from 10 camera point clouds by PSR.

When global mesh *M* is fixed then the optimization process becomes aligning only one camera position to the global mesh. Optimizing all camera poses in Equation (2) becomes optimizing each camera pose in Equation (7):

(7) $$E_{i}\left(T\right)=\sum_{p\in P_{i}}\left(D\left(M,p\right)\right)+\Gamma \left(T_{i},T_{i0}\right)$$

Each objective function $E_{i}\left(T\right)$ contains only the six variables $\xi_{i}$ . In each iteration, the Gaussian–Newton method is performed with six variables.
**Algorithm 1** Algorithm of alignment for all cameras
  1:**for** i = 1 to 10 **do**  2:    initial camera i pose from calibration parameters.  3:**end for**  4:**for** i = 1 to 10 **do**  5:    roughly compute the overlapping between camera i with other cameras  6:**end for**  7:**repeat**  8:    computes global mesh from 10 cameras by PSR  9:    **for** i = 1 to 10 **do**10:        align mesh i to global mesh11:    **end for**12:**until** residual difference ≤ threshold value13:generates final mesh by PSR algorithm


## 5. Experimental Results

In this section, the hardware specifications and the outcomes of cattle reconstruction are provided. Additionally, the distinctions in cattle reconstruction results, compare cases with and without global optimization. Furthermore, we document the reduction in errors during the process of global optimization.

### 5.1. Evaluation of Synchronization Process

**Figure 5:**
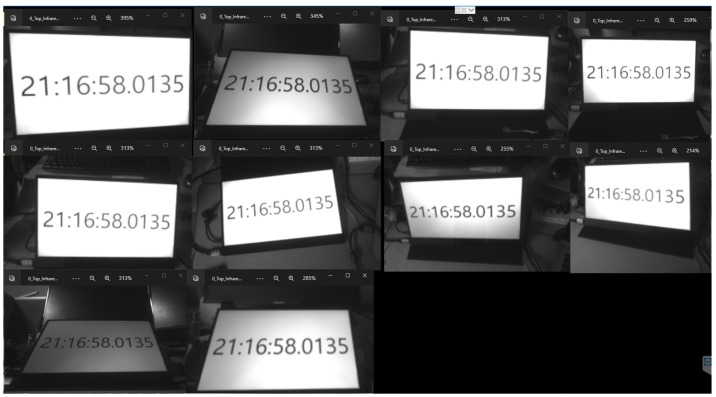
Synchronization of camera system.

To assess the synchronization between the 10 cameras in the 3D reconstruction system, we generated a capture signal to capture images of the ten cameras. The synchronization was demonstrated through the frames obtained from the ten cameras, which displayed the same timestamp on the screen Figure 5.

### 5.2. Evaluation of Optimization Process

To compare the optimization result of our optimization method we used an up-to-date global optimization—the BCMO algorithm. Computation time by the global optimization method—BCMO—is slow, so a small data set of only three cameras was optimized for this comparison.

**Figure 6:**
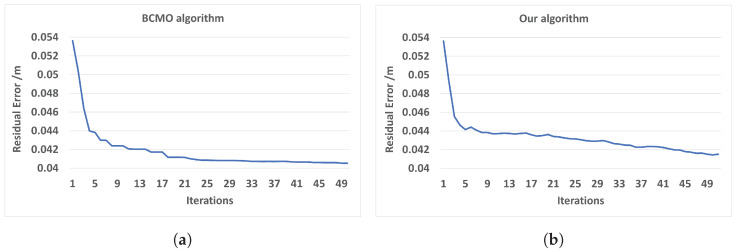
Residual error for 3-camera optimization. (**a**) BCMO algorithm. (**b**) Our optimization.

For optimizing three camera poses, each camera had six parameters and one camera was fixed. Therefore, there were a total of 12 parameters optimized. For six variables of the BCMO algorithm, the translation variable was set in the range of −0.1 m to 0.1 m, and the angle was set in the range of −3 to 3 degrees. In the BCMO algorithm, the parameter population was set to 1000, and the parameter iteration was set to 50. Figure 6 shows that for global optimization with all variables, the error result of method BCMO was a little better than the result of our method, but the computation time was much slower (Table 2). The optimization spends most of its time working on finding correspondence points between clouds.

**Figure 7:**
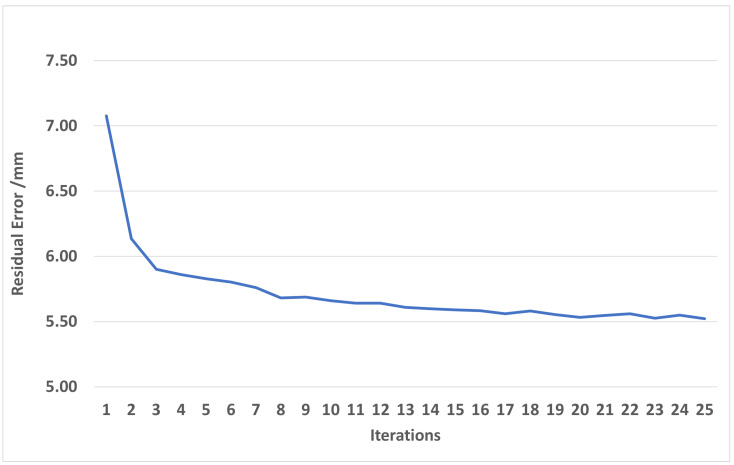
Residual error between input data and output model M.

In the next experiment, the residual error was computed after the alignment of 10 cameras. From the dataset of 10 cameras, model mesh M was generated. Model M has ∼72.000 vertices and ∼144.000 triangles. The total overlapping points of 10 cameras is about ∼1.9 million points. When we generated the mesh by the PSR algorithm from points and normals, we set the option depth to 8 to create a smooth mesh, M. Then, we used model M to align each camera as overlapping. A final output mesh was created by the PSR algorithm with an option depth of nine to get more details on its surface. The computation time for each iteration includes the time of two parts: the time for mesh reconstruction by the PSR algorithm is about 5 s; the time for camera pose optimization is about 6 s. The total time for 1 iteration is about 11 s. The mean error point on the 10-camera mesh and model M reduces from 7.5 mm to 5.5 mm after 25 iterations. The results from Figure 6 and Figure 7 have large differences due to several reasons. The first reason is that in Figure 6, the residual is computed based on point-to-point distances, and in Figure 7, the distance is computed based on point-to-plane distances. The distance between two clouds is computed based on the point-to-plane being smaller than point-to-point; it depends on point resolution. The second reason is that in Figure 7, the residual is between points, and mesh M is approximated to the average of all frames, so roughly, it is approximately equal to half of the distance between frames. The last reason is that when optimizing the objective function, all points with a distance smaller than 0.14 to other mesh points are also valid points.

### 5.3. Cow Reconstruction Result

**Figure 8:**
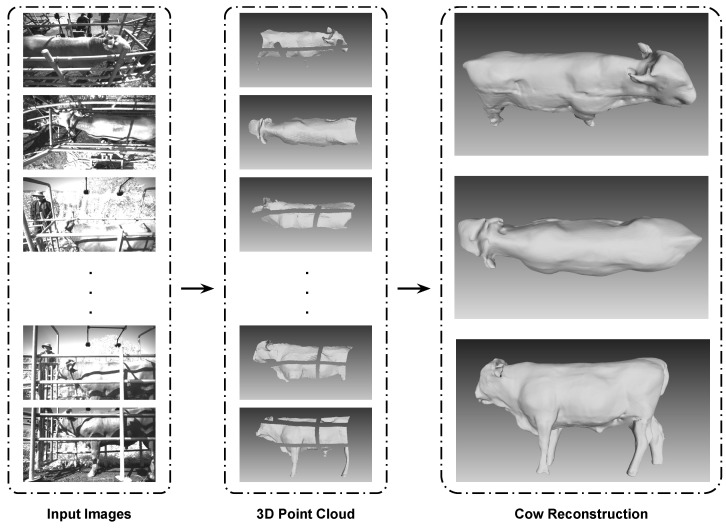
Cow reconstruction process.

**Figure 9:**
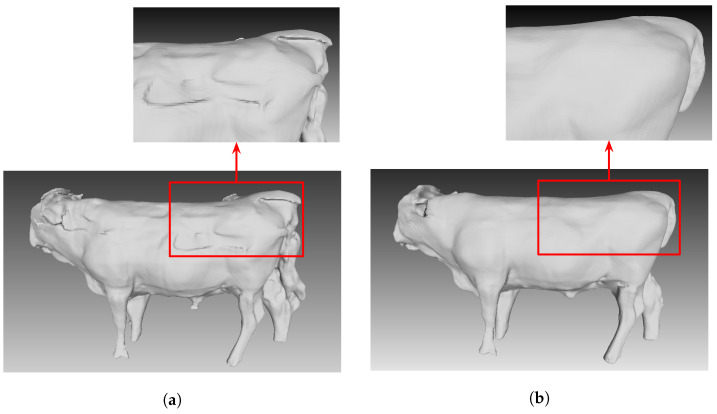
The reconstruction output. (**a**) Before global optimization. (**b**) After global optimization.

In Figure 8, our results show we can align all ten cameras to create a final cow reconstruction model. Additionally, the cattle surface, which is invisible by a fence on one camera view, can be filled in by another camera view or can be filled in by the PSR algorithm. Based on both residual value and computation time, using the method of interleaving update is a practical method. Figure 9 shows the mesh surface before global optimization (Figure 9a) and after global optimization (Figure 9b).

## 6. Conclusions

In this paper, an automatic process from capturing data to generating a 3D mesh is presented. Experimental results with actual Korean cattle data demonstrate that the mesh output has quality almost equivalent to an up-to-date global optimization algorithm but the time to reconstruction is much faster. When the number of cameras increases, our algorithm computation time linearly increases from a high-quality mesh output of Korean cattle, and Korean cattle’s features can easily be analyzed or the mesh can be used as an input database for AI research. In this research, the light issue is not mentioned and the reconstruction process fails if the input data (stereo images) are too bright. The light issue needs to be analyzed more in the future. The computation time of BCMO depends on the number of population and iteration parameters, to decrease computation time, using GPU is a potential method to speed up computation time. To improve output mesh precision, the camera distortion should be considered. However, in this camera system, 10 cameras have different distortion models, and pre-calibrating distortion models of each camera can improve results in the future.

## Figures and Tables

**Table 1:** The specifications of the hardware device.

Device	Specification
Depth Camera (Intel Realsense D435i)	- Use environment: Indoor/Outdoor - Baseline [mm]: 50 - Resolution: 1920 × 1080 px - Frame rate: 30 fps - Sensor FOV (H × V × D): 69.40 × 42.5 × 77 (±3) - Dimensions: 90 × 25 × 25 mm - Connection: USB-C 3.1 Gen1
Single Board Computer (Jetson Nano)	- GPU: 128-Core Maxwell - CPU: Quad-core ARM Cortex-A57 CPU - RAM: 4 GB 64-bit LPDDR4 25.6 GB/s - Storage: microSD card slot for storage (256 GB) - USB: 4× USB 3.0 ports, USB 2.0 Micro-B - Networking: Gigabit Ethernet - Wireless: Optional Wi-Fi/Bluetooth module - Operating System: Supports NVIDIA’s Linux-based operating system - Power: 5 V/4 A power supply
Host Computer (Jetson Orin)	- GPU: 2048-core NVIDIA Ampere architecture GPU with 64 Tensor Cores - CPU: 12-core Arm^®^ Cortex^®^-A78AE v8.2 64-bit, CPU - RAM: 64 GB 256-bit LPDDR5, 204.8 GB/s - Storage: 64 GB eMMC 5.1 storage - USB: Up to 2 × 8, 1 × 4, 2 × 1 (PCIe Gen4, Root Port and Endpoint), 3 × USB 3.2 - Networking: 1 × GbE, 1 × 10 GbE - Operating System: Supports various Linux distributions - Power: 15 W–60 W

**Table 2:** Computation time for 3-camera optimization by BCMO algorithm and our algorithm.

	Number of Iteration	1 Iteration Computation Time	Total Time
BCMO Algorithm	50 iterations	280 s	14,000 s
Our algorithm	50 iterations	11 s	550 s

## Data Availability

The data presented in this study are available on request from the corresponding author.

## Abbreviations

The following abbreviations are used in this manuscript:

3D	Three Dimension
A.I	Artificial Intelligence
BCMO	Balance Composite Motion Optimization
FPFH	Fast-Point Feature Histogram
ICP	Iterative Closest Point
Kd-tree	K-dimension tree
PSR	Poisson Surface Reconstruction
M	3D Mesh generated from points and normals of 10 cameras by PSR algorithm
Super4PCS	Super 4-Point Congruent Set
T	Transformation matrix

## References

[1] Özyeşil O., Voroninski V., Basri R., Singer A. (2017). A survey of structure from motion. Acta Numer..

[2] Newcombe R.A., Izadi S., Hilliges O., Molyneaux D., Kim D., Davison A.J., Kohi P., Shotton J., Hodges S., Fitzgibbon A. Kinectfusion: Real-time dense surface mapping and tracking. Proceedings of the 2011 10th IEEE International Symposium on Mixed and Augmented Reality.

[3] Zhou Q.Y., Koltun V. Simultaneous localization and calibration: Self-calibration of consumer depth cameras. Proceedings of the IEEE Conference on Computer Vision and Pattern Recognition.

[4] Besl P.J., McKay N.D. Method for registration of 3-D shapes. Proceedings of the Sensor Fusion IV: Control Paradigms and Data Structures, Spie.

[5] Segal A., Haehnel D., Thrun S. Generalized-icp. Proceedings of the Robotics: Science and Systems.

[6] Horn B.K. (1987). Closed-form solution of absolute orientation using unit quaternions. J. Opt. Soc. Am. A.

[7] Zhang Z. (1994). Iterative point matching for registration of free-form curves and surfaces. Int. J. Comput. Vis..

[8] Aiger D., Mitra N.J., Cohen-Or D. (2008). 4-points congruent sets for robust pairwise surface registration. ACM SIGGRAPH 2008 Papers.

[9] Rusu R.B., Blodow N., Beetz M. Fast point feature histograms (FPFH) for 3D registration. Proceedings of the 2009 IEEE International Conference on Robotics and Automation.

[10] Zhang J., Singh S. LOAM: Lidar odometry and mapping in real-time. Proceedings of the Robotics: Science and Systems.

[11] Wang S., Zuo X., Du C., Wang R., Zheng J., Yang R. (2018). Dynamic non-rigid objects reconstruction with a single rgb-d sensor. Sensors.

[12] Newcombe R.A., Fox D., Seitz S.M. Dynamicfusion: Reconstruction and tracking of non-rigid scenes in real-time. Proceedings of the IEEE Conference on Computer Vision and Pattern Recognition.

[13] Le-Duc T., Nguyen Q.H., Nguyen-Xuan H. (2020). Balancing composite motion optimization. Inf. Sci..

[14] Choi S., Zhou Q.Y., Koltun V. Robust reconstruction of indoor scenes. Proceedings of the IEEE Conference on Computer Vision and Pattern Recognition.

[15] Park J., Zhou Q.Y., Koltun V. Colored point cloud registration revisited. Proceedings of the IEEE International Conference on Computer Vision.

[16] Ruchay A., Kober V., Dorofeev K., Kolpakov V., Gladkov A., Guo H. (2022). Live Weight Prediction of Cattle Based on Deep Regression of RGB-D Images. Agriculture.

[17] Li J., Ma W., Li Q., Zhao C., Tulpan D., Yang S., Ding L., Gao R., Yu L., Wang Z. (2022). Multi-view real-time acquisition and 3D reconstruction of point clouds for beef cattle. Comput. Electron. Agric..

[18] Li S., Lu R., Liu J., Guo L. (2021). Super edge 4-points congruent sets-based point cloud global registration. Remote Sens..

[19] Bueno M., Bosché F., González-Jorge H., Martínez-Sánchez J., Arias P. (2018). 4-Plane congruent sets for automatic registration of as-is 3D point clouds with 3D BIM models. Autom. Constr..

[20] Le Cozler Y., Allain C., Caillot A., Delouard J., Delattre L., Luginbuhl T., Faverdin P. (2019). High-precision scanning system for complete 3D cow body shape imaging and analysis of morphological traits. Comput. Electron. Agric..

[21] Rusinkiewicz S., Levoy M. Efficient variants of the ICP algorithm. Proceedings of the Third International Conference on 3-D Digital Imaging and Modeling.

[22] Li J., Wang P., Xiong P., Cai T., Yan Z., Yang L., Liu J., Fan H., Liu S. Practical stereo matching via cascaded recurrent network with adaptive correlation. Proceedings of the IEEE/CVF Conference on Computer Vision and Pattern Recognition.

[23] Dang C., Choi T., Lee S., Lee S., Alam M., Lee S., Han S., Hoang D.T., Lee J., Nguyen D.T. (2022). Case Study: Improving the Quality of Dairy Cow Reconstruction with a Deep Learning-Based Framework. Sensors.

[24] Staranowicz A.N., Brown G.R., Morbidi F., Mariottini G.L. (2015). Practical and accurate calibration of RGB-D cameras using spheres. Comput. Vis. Image Underst..

[25] Kazhdan M., Bolitho M., Hoppe H. Poisson surface reconstruction. Proceedings of the Fourth Eurographics Symposium on Geometry Processing.

[26] Kazhdan M., Hoppe H. (2013). Screened poisson surface reconstruction. ACM Trans. Graph. (ToG).

